# Patterns of *Dual-Specific Phosphatase 4* mRNA Expression Before and after Neoadjuvant Chemotherapy in Breast Cancer

**DOI:** 10.31557/APJCP.2019.20.4.1051

**Published:** 2019

**Authors:** Prihantono Prihantono, Andi Nilawati Usman, Christian Binekada, Mochammad Hatta, Andi Asadul Islam

**Affiliations:** 1 *Department of Surgery, *; 4 *Biology Molecular and Immunology Laboratory, Faculty of Medicine, *; 2 *Department of Statistics, Faculty of Health Community, Hasanuddin University, Makassar,*; 3 *Department of Surgery, Faculty of Medicine, Haluoleo University, Kendari, Indonesia. *

**Keywords:** DUSP4, mRNA, qRT-PCR, Chemotherapy response

## Abstract

**Objective:**

Evaluation of the neoadjuvant chemotherapy response can be performed by comparing the breast cancer burden and pathobiology before and after treatment. This study was aimed to investigate the pattern of dual-specific phosphatase 4 (*DUSP4*) mRNA expression in breast cancer cells before and after neoadjuvant chemotherapy.

**Methods:**

This was a longitudinal study. Twenty samples of matched breast cancer tissue taken from biopsy before and after chemotherapy were subjected to qRT-PCR to detect *DUSP4* mRNA expression.

**Results:**

The mean value of *DUSP4* mRNA expression in prechemotherapy breast cancer patients was 9.906±0.333 and that in breast cancer patients postchemotherapy was 10.016±1.062. In the responsive group, the rate of *DUSP4* mRNA expression increased by 0.476 after chemotherapy. In the nonresponsive group, the proportion of *DUSP4* mRNA expression likely decreased by 1.012. Statistical analysis found no significant correlation between *DUSP4* mRNA expression prechemotherapy and the clinical chemotherapeutic response with p-value = 0.994 (p ≥0.05). A significant correlation was found between the postchemotherapy *DUSP4* mRNA expression and the clinical chemotherapeutic response with p-value = 0.003 (p < 0.5).

**Conclusion:**

No significant difference was found in the mRNA expression of *DUSP4* in pre- and post-neoadjuvant chemotherapy specimens. High *DUSP4* expression postchemotherapy shows a substantial correlation with the chemotherapeutic response.

## Introduction

Breast cancer is the most common cancer and the second leading cause of cancer death in women worldwide, including Indonesia (Bray et al., 2013; Youlden et al., 2014). Advances in systemic therapy, such as chemotherapy, hormonal therapy, and targeting treatment, have improved the patient disease-free survival and overall survival, but some cancers are resistant to systemic therapy (Martin et al., 2014; Miller et al., 2016). Cancer patients with the same stage, grade, and histogenesis can have different treatment responses to various chemotherapy agents (Luqmani, 2005; Rouzier et al., 2005; Györffy et al., 2006). Some theories have proposed the mechanisms of therapeutic resistance. Biomarker and gene-specificity for chemotherapeutic resistance are challenges to be addressed (Holohan et al., 2013).

Neoadjuvant chemotherapy offers an estimation of the treatment response (Cortazar et al., 2014; Zardavas and Piccart, 2015). Chemotherapy induces upregulation or downregulation of most genes (Klintman et al., 2016). Residual disease after neoadjuvant chemotherapy may predict the prognosis and gene expression in residual disease, suggesting a biologic role in chemoresistant disease (Balko et al., 2012; Klintman et al., 2016) (Penault-Llorca and Radosevic-Robin, 2016).

Dual-specificity phosphatase 4 (*DUSP4*) is a protein responsible for dephosphorylating threonine/serine and tyrosine residues on their substrates (Boulding et al., 2016). *DUSP4* selectively dephosphorylates signaling Mitogen-activated protein kinase’s (MAPKs), implicating them in signal transduction. Studies have found that *DUSP4* is upregulated in malignant tissues, including breast cancer (Boulding et al., 2016).

Mazumdar et al., (2016) identified the low expression of the *DUSP4* protein in ER-negative breast cancers. Overexpression of *DUSP4* protein causes dephosphorylation of growth-promoting signaling proteins, hence inhibiting the growth and invasiveness of ER-negative breast cancer cells.

*DUSP4* is signiﬁcantly enriched in response to chemotherapy, and low levels of *DUSP4* in residual disease are associated with an impaired prognosis (Klintman et al., 2016). The loss of *DUSP4* activates the MAPK pathway, promoting a stem cell-like phenotype and decreasing the clinical response to neoadjuvant therapy in breast cancer (Balko et al., 2012; Balko et al., 2013). Some other studies also found that *DUSP4* expression is associated with resistance to cytotoxic chemotherapies such as doxorubicin and cisplatin chemoresistance (Liu et al., 2013; Boulding et al., 2016).

The objective of this study was to determine the pattern of *DUSP4* mRNA expression in locally advanced breast cancer patients pre- and post-neoadjuvant chemotherapy using anthracycline-based chemotherapy and the relationship with the clinical chemotherapeutic response.

## Materials and Methods

*Samples*

This was an observational study. The samples were obtained from Wahidin Sudirohusodo Hospital Makassar, a top referral hospital in the east of Indonesia, from February to June 2016. Female patients with locally advanced breast cancer, invasive ductal carcinoma type, receiving neoadjuvant chemotherapy with a cyclophosphamide-adriamycin-5-FU regimen, were included in the study. The DUSP4 mRNA expression was detected using quantitative real-time polymerase chain reaction (qRT-PCR) from breast cancer tissue taken from biopsy and surgery.

*Nucleic Acid Extraction*

Samples of breast cancer tissue were subjected to nucleic acid extraction using the Boom method (diatom guanidinium isothiocyanate (GuSCN) method). Breast cancer tissue as much as 100 µg/ul was added to 900 mL “L6” solution containing 120 g GuSCN, 100 ml 0.1 mM Tris-HCl, pH 6.4, 22 ml 0.2 mM ethylenediaminetetraacetate (EDTA), pH 8.0, and 2.6 g Triton X-100 (final concentrations of 50 mM Tris- HCl, 5 M GuSCN, 20 mM EDTA, and 0.1% Triton X-100). Next, 20 ml diatom suspension was added consisting of 50 ml H_2_O and 500 mL 32% (w/v) Diatoms. This diatom suspension, which could bind 10 µg DNA tissue, was vortexed and centrifuged in a 1.5-ml Eppendorf tube at 13,000 rpm for 15 seconds. The supernatant was removed, and the sediment was washed with 1 ml “L2” solution (120 g GuSCN in 100 ml 0.1 M Tris-HCl, pH 6.4). Next, the sample was vortexed and then centrifuged at 13,000 rpm for 15 seconds. Next, the sediment was washed twice with “L2” solution and twice with 1 ml 70% ethanol and 1 ml acetone. The sample was then heated in a water bath at a temperature of 56°C for 10 minutes, followed by the addition of 60 mL “TE” solution (1 mM EDTA in 10 mM Tris-HCl, pH 8.0), vortexing and centrifugation at 13,000 rpm for 2 minutes. The sample was then incubated in an oven at 56°C for 10 minutes, followed by vortexing and centrifugation at 13,000 rpm for 30 seconds and collection of the supernatant. This supernatant was the result of nucleotide extraction and was stored at −80°C before PCR analysis (Boom et al., 1990; Prihantono et al., 2017).

*Expression of mRNA DUSP4 Genes by Real-Time PCR*

The detection of *DUSP4* mRNA expression was performed according to real-time PCR as described by Liu et al. (Liu et al., 2013). The specific primers for *DUSP4* mRNA were as follows: forward, 5′- CCCACAGAGCAGTATTAGGCTGAAG-3′; reverse, 5′-CAGCGTGGATGAGCAACTGAA-3′. The primers for the reference gene (*β-actin* gene) were as follows: forward, 5′-GGAGATTACTGCC- CTGGCTCCTA-3′; reverse, 5′- GACTCATCGTACTCCTGCTTGCTG-3′. Each sample required 1 μg of the template. Reverse transcription was performed using the RT reagent Kit with gDNA eraser. cDNA was synthesized using the cDNA Synthesis kit (Takara) following the instructions provided by the manufacturers. qRT-PCR was performed using an ABI7500 Sequence Detection System (PE Applied Biosystems) in the presence of SYBR-green I. Briefly, a 50-μl reaction mix containing 25 μl Premix ExTaq , Takara), 1 μl ROX reference Dye II ( Takara), 1 μl PCR forward primer (10 μM), 1 μl PCR reverse primer (10 μM), 4 μl cDNA and 18 μl dH_2_O was premixed before reaction in 96-well plates. The reaction protocol was as follows: 95°C for 30 s, 40 cycles of 95°C for 5 s and 60°C for 34 s, followed by 95°C for 15 seconds, 60 °C for 1 minute and 95°C for 15 seconds. The relative gene expression profiles were determined by normalizing to the reference gene (β-actin) using the 2_ΔCt method. Each sample for this study was tested in triplicate (Liu et al., 2013).

*Chemotherapeutic Response Criteria*

The response to neoadjuvant chemotherapy was classified according to RECIST criteria. The nonresponsive group displayed stable disease or progressive disease according to RECIST criteria if there is a reduction of the tumor size less than 30%, no change, an increase in the tumor size, or a new tumor. The responsive groups displayed a complete or partial response if there is a reduction of the tumor size >30%, no evidence of a tumor clinically or pathologically, or no further tumor found.

*Ethical Clearance*

This study has been approved by the Ethical Commission of Health Study, Medical Faculty, Hasanuddin University, with the registry number 799/H4.8.4.5.31/PP36-KOMETIK/2016 (Register: UH15060492).

## Results

*Characteristics*

**Figure 1:**
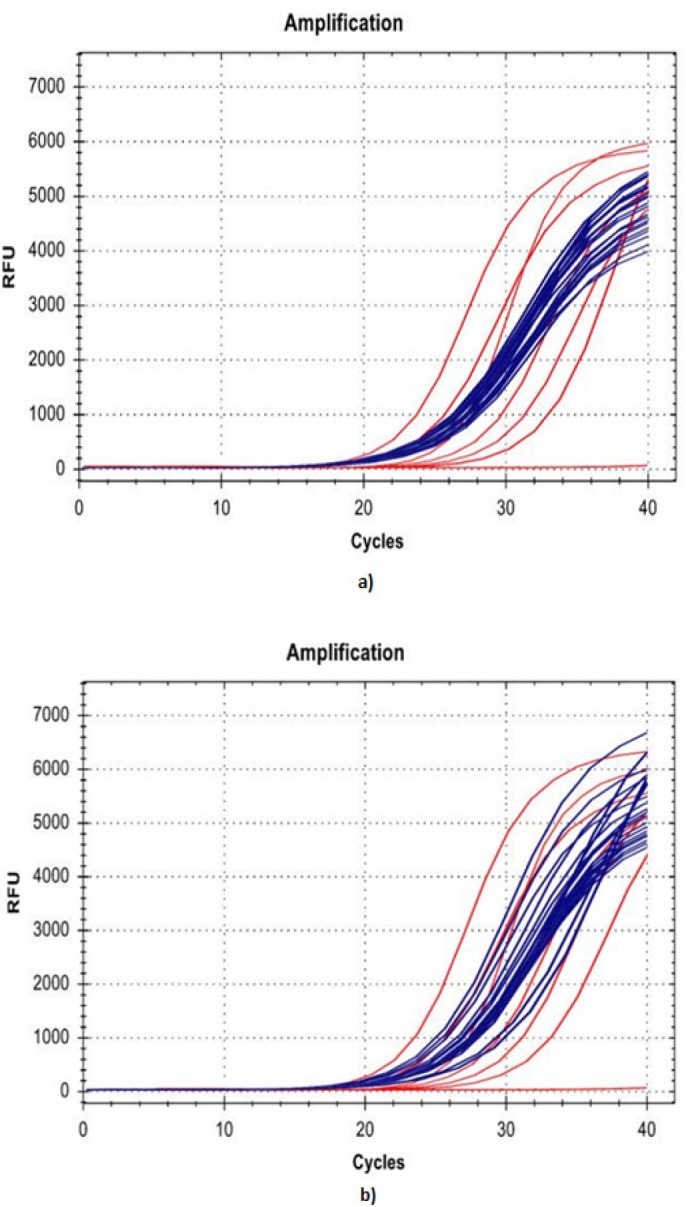
Amplification Curve of *DUSP4*: a) Prechemotherapy; b) Postchemotherapy

The twenty enrolled female patients with invasive breast carcinoma diagnosed and treated at Wahidin Sudirohusodo General Hospital met the inclusion criteria of the study. Their ages ranged from 28 to 64 years, with a mean age of 50.3 years. All twenty cases were invasive ductal carcinoma. The obtained histopathologic grading was a low grade in 1 case (5%), moderate grade in 15 cases (75%) and high grade in 4 cases (20%). The number of patients responsive to neoadjuvant chemotherapy was 15/20 (75%), and the number of nonresponsive patients was 5/20 (25%). No correlation was found between *DUSP4* mRNA expression and clinical data, including age and grade. The characteristics of the samples are presented in Table 1, and the amplification curve of *DUSP4* prechemotherapy and postchemotherapy is shown in Figure 1.

**Table 1:** Characteristics of Samples

Characteristic	n (%)	p*
Age		0.617
≤ 50 years	11 (55.0%)	
> 50 years	9 (45.0%)	
Grade		0.225
Low Grade	1 (5.0%)	
Moderate Grade	15 (75.0%)	
High Grade	4 (20.0%)	
Immunohistochemistry		0.56
ER	5 (25.0%)	
PR	6 (30.0%)	
HER2	13 (65.0%)	
Ki-67	11 (55.0%)	
Clinical response		0.959
Luminal A	3 (15.0%)	
Luminal B	6 (30.0%)	
HER2	7 (35.0%)	
Triple Negative	4 (20.0%)	
Clinical response		
Responsive	15 (75.0%)	
Nonresponsive	5 (25.0%)	

*p, chi-squared test for the clinical chemotherapy response

**Table 2:** Comparison of the mRNA *DUSP4* Expression Pre- and Postchemotherapy with the Clinical Response

mRNA Expression	Responsive (n=20)	Non Responsive (n=7)	Mean difference p-value
*DUSP4* (Prechemotherapy)	9.902±0.336	9.917±0.378	-0.015*
*DUSP4* (Postchemotherapy)	10.378±0.785	8.905±1.082	1.472**
Mean difference	-0.476	1.012	

Source: Primary Data, *Independent Samples T-test, **Mann-Whitney U test

**Table 3:** Correlation of the mRNA *DUSP4* Expression and the Clinical Chemotherapeutic Response

	mRNA Expression	Correlation with the	p
	(Mean±SD)	Chemotherapy response	
*DUSP4* mRNA (Prechemotherapy)	9.906±0.333	-0.002	0.994*
*DUSP4* mRNA (Postchemotherapy)	10.016±1.062	0.494	0.027**
Rate of DUSP4 mRNA Expression	1.329±1.283	0.24	0.307**

Source: Primary Data, * Pearson, ** Spearman

Comparison of the mRNA *DUSP4* expression pre- and postchemotherapy is shown in Table 2. The mean value of *DUSP4* mRNA expression in breast cancer patients prechemotherapy who were responsive to chemotherapy was 9.902±0.336, whereas that of nonresponsive patients was 9.917±0.378. The mean value of *DUSP4* mRNA expression postchemotherapy in those sensitive to chemotherapy was 10.378±0.785, whereas that of nonresponsive patients was 8.905±1.082. In the sensitive group, *DUSP4* mRNA expression increased by 0.476. In the nonresponsive group, the *DUSP4* mRNA expression decreased by 1.012. No significant difference was found in the mean *DUSP4* mRNA expression value between the prechemotherapy group and the group with clinical response to chemotherapy (p-value = 0.939; p ≥0.05). A significant mean difference was found between *DUSP4* mRNA expression postchemotherapy and the clinical response to chemotherapy (p-value = 0.003; p ≥0.05). 

The relationship between the *DUSP4* mRNA expression and the clinical response is demonstrated in Table 3. No significant correlation was found between the mRNA expression of *DUSP4* prechemotherapy with the clinical response (p = 0.994; p>0.05). A definite correlation was found between *DUSP4* mRNA expression postchemotherapy and the clinical response (r = 0.494); this association was significant with p = 0.027 (p<0.05). A definite correlation was found between the rate of *DUSP4 *mRNA expression and the clinical response with a value of r = 0.240; this association was insignificant with p = 0.307 (p>0.05).

## Discussion

Breast cancer chemo-resistance influenced by several factors including drug inactivation, changes in drug targets, overexpression of ABC transporters, apoptotic dysregulation, epigenetic regulation, epithelial to mesenchymal transition, and cancer stem cells (Housman et al., 2015).

*DUSP4* expression has been found in various human cancers (Kidger and Keyse, 2016). Over-expression of *DUSP4* is frequently observed in breast cancer and may play an essential role in cancer development and progression (Wang et al., 2003).* DUSP4* is commonly upregulated in breast malignancy and may play a crucial role in cancer development and progression. *DUSP4* may be a marker of adverse prognosis, especially in patients with early breast cancer (Kim et al., 2015). 

Decreased expression of *DUSP4*, a negative regulator of extracellular signal-regulated kinases (ERK), is related to high RAS-ERK activation and has been recently identified as a mediator of resistance to neoadjuvant chemotherapy in triple-negative breast cancer, promoting to a shorter recurrence-free survival (Balko et al., 2012; Rottenberg and Jonkers, 2012). *DUSP4* expression was also found to be responsible for the resistance to etoposide and mitoxantrone chemotherapy in breast cancer (Györffy et al., 2006).

We found that increased* DUSP4* mRNA expression showed a better chemotherapy response than decreased *DUSP4* mRNA expression, but the difference was not statistically significant. *DUSP4* mRNA expression postchemotherapy was associated with chemotherapy response.

In our previous study on the expression of *DUSP4 *using immunohistochemistry, *DUSP4* expression was found in 33% (21/63) of breast cancer samples. Analysis of *DUSP4* expression with a chemotherapy response found no significant correlation, with p = 0.073 (> 0.05). However, stratification of *DUSP4* expression based on the intrinsic subtype found that the Luminal B p-value = 0.02 (<0.05), the Luminal A p-value = 0.24 (> 0.05), and the Her2 p-value = 0.608 (> 0.05); the triple-negative subtype could not be analyzed because of the small number of samples. Furthermore, *DUSP4* expression was correlated with the anthracycline-based chemotherapy response in the luminal B subtype (Prihantono et al., 2017).

Balko et al., (2012) found that low *DUSP4* expression was associated with basal-like breast cancer, high tumor proliferation after chemotherapy, and a decrease in the clinical chemotherapeutic response and achieved poorer pathologic complete remission rates and shorter recurrence-free survival periods than those in patients with high levels of *DUSP4* expression. By contrast, *DUSP4* overexpression was associated with increased apoptosis induced by chemotherapy (Balko et al., 2012). 

Baglia et al., (2014), in his study, found that low *DUSP4* expression was associated with increased recurrence and mortality in triple-negative breast cancer patients. Baglia concluded that low *DUSP4* expression is a predictor of recurrence and death in triple-negative breast cancer patients.

Liu et al., (2013) demonstrated that *DUSP4* expression affects the breast cancer cell response to chemotherapy. High *DUSP4* expression requires higher doses of doxorubicin, whereas cells with low *DUSP4* expression need lower doses of doxorubicin. Doxorubicin chemotherapy in breast cancer cells with high *DUSP4* expression can lead to acquired chemoresistance by converting epithelial cells into mesenchymal (EMT) cells (epithelial-to-mesenchymal transition). With these EMT changes, cancer cells become more actively proliferating, invasion, migration, and apoptosis are reduced, and the cells become less sensitive to chemotherapy. 

Hae Hyun Jung (2016), suggest that the loss of *DUSP4*, a potential biomarker of treatment-resistant TNBC, is associated with ets-1 overexpression via the PI3K and MAPK pathways. Statin, a small inhibitor of HMG-CoAR, is a likely therapeutic candidate for treatment-resistant TNBC because it can reverse ets-1 overexpression by restoring *DUSP4* expression.

This study found *DUSP4* mRNA expression postchemotherapy was associated with chemotherapy response. This finding is in line with the article of Rottenberg and Jonkers (2012), Cells with low *DUSP4 *expression show a high Ki-67 score, which is associated with a poor long-term outcome after neoadjuvant chemotherapy. Hence, the residual cells that show low *DUSP4* expression are not quiescent, drug-tolerant cells. Instead, they appear to be genuinely drug-refractory and proliferate regardless of drug treatment. Residual cancer cells may still have another backup: entering a quiescence programme and lying low until the drug is eliminated (Rottenberg and Jonkers, 2012).

In conclusion, no significant difference was found in the *DUSP4* mRNA expression of pre- and post-neoadjuvant chemotherapy specimens. Increased *DUSP4* mRNA expression shows the tendency of better chemotherapy response, but it is not statistically significant. These results do not suggest that* DUSP4* mRNA expression plays a role in conferring neoadjuvant chemotherapy resistance. *DUSP4 *expression postchemotherapy has a substantial correlation with the chemotherapy response. The findings warrant further research to observe the disease-free survival and overall survival with a larger sample size.

## Grant Support

This study was supported by funds from the Ministry of Health of the Republic of Indonesia through a research grant for novice researchers entitled “RISBINIPTEKDOK 2016”.

## Conflicts of Interest

We have no conflicts of interest.
